# Ultrastable tip-enhanced hyperspectral optical nanoimaging for defect analysis of large-sized WS_2_ layers

**DOI:** 10.1126/sciadv.abo4021

**Published:** 2022-07-15

**Authors:** Ryo Kato, Toki Moriyama, Takayuki Umakoshi, Taka-aki Yano, Prabhat Verma

**Affiliations:** ^1^ Department of Applied Physics, Osaka University, Suita, Osaka 565-0871, Japan.; ^2^ Institute of Post-LED Photonics, Tokushima University, 2-1 Minamijosanjima, Tokushima, Tokushima 770-8506, Japan.; ^3^ Institute for Advanced Co-Creation Studies, Osaka University, Suita, Osaka 565-0871, Japan.; ^4^ PRESTO, Japan Science and Technology Agency, Kawaguchi, Saitama 332-0012, Japan.

## Abstract

Optical nanoimaging techniques, such as tip-enhanced Raman spectroscopy (TERS), are nowadays indispensable for chemical and optical characterization in the entire field of nanotechnology and have been extensively used for various applications, such as visualization of nanoscale defects in two-dimensional (2D) materials. However, it is still challenging to investigate micrometer-sized sample with nanoscale spatial resolution because of severe limitation of measurement time due to drift of the experimental system. Here, we achieved long-duration TERS imaging of a micrometer-sized WS_2_ sample for 6 hours in a reproducible manner. Our ultrastable TERS system enabled to reveal the defect density on the surface of tungsten disulfide layers in large area equivalent to the device scale. It also helped us to detect rare defect-related optical signals from the sample. The present study paves ways to evaluate nanoscale defects of 2D materials in large area and to unveil remarkable optical and chemical properties of large-sized nanostructured materials.

## Teaser

Long-duration nanoscale Raman imaging was demonstrated for defect analysis of large-sized nanostructured materials.

## INTRODUCTION

Optical spectroscopic techniques, such as Raman spectroscopy, are arguably the best nondestructive tools to characterize and study the intrinsic properties of a variety of samples. However, the diffraction limit of light does not allow one to probe a sample at a spatial resolution better than about half of the probing wavelength. By involving near-field optical microscopy in Raman spectroscopy, a technique known as the tip-enhanced Raman spectroscopy (TERS) (*1*–*6*), one can break this limit and achieve true nanometric spatial resolution in optical microscopy. A similar near-field technique for nanoscale optical imaging is the tip-enhanced photoluminescence (TEPL) (*7*–*9*). In both these techniques, a metallic nanotip is used to enhance and confine the light field within a nanometric volume in the vicinity of the tip apex through plasmon resonance, and the sample is probed through this confined light (*10*, *11*). This has opened the way for optical investigation of different samples at the spatial resolution of a few nanometers, far beyond the diffraction limit of the light (*12*–*14*). Numerous applications of TERS and TEPL for optical nanoinvestigation of various samples have been demonstrated in a wide range of fields, for instance, probing topographically hidden nanoscale defects, grain boundaries and other imperfections in two-dimensional (2D) materials (*15*–*20*), spectroscopic visualization of heterogeneous catalytic sites (*21*, *22*), and single-molecule detection and imaging (*23*–*25*). Furthermore, hyperspectral optical nanoimaging, which is essentially a combination of TERS and TEPL, has attracted increasing attention for these applications, because it allows a direct correlation of nanoscale topography with chemical states and distinct optical properties of a material, such as bandgap of the 2D materials and plasmon-molecule interactions (*26*–*28*).

**Fig. 1.**
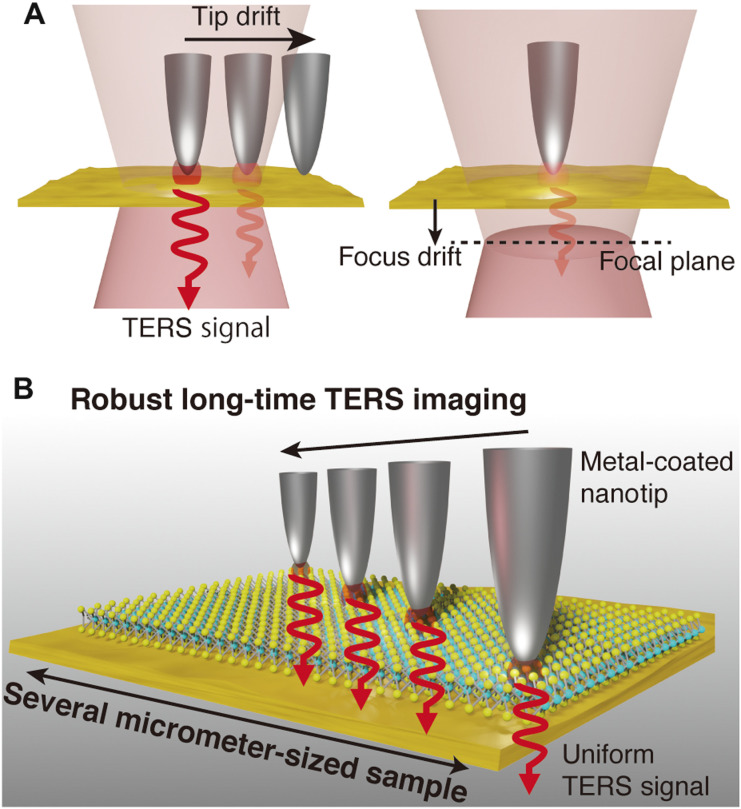
Long-duration TERS imaging with drift compensation techniques. (**A**) (Left) Time-dependent drift of the relative position between a metallic tip and the laser spot in the lateral direction and (right) along the optical axis. Both time-dependent tip drift and focus drift degrade enhanced optical field at the tip apex. (**B**) Schematic illustration of stable nano-optical imaging from a large area of WS_2_ monolayer placed on a gold thin film that does not degrade the TERS signal for a long duration.

While TERS has found its innovative multidimensional nanoscale applications in the recent past, it has a vital glitch in imaging large-sized samples, even for samples with the size of a few micrometers. It is often a challenging task to maintain stable TERS signal for a large sample through the entire measurement period, especially when using an atomic force microscopy (AFM)–based TERS setup. The instability of the scattered signal originates from thermal and vibrational drift of the metallic nanotip with respect to the focus spot of the incident light. In TERS measurement, a metallic nanotip should be positioned at the center of the focus spot of the excitation laser to effectively excite the localized surface plasmon. To ensure stable scattered signal throughout the measurement, the relative position of the nanotip and the focus spot must remain locked during the entire imaging process. However, because of a possible drift of the system due to thermal and vibrational fluctuations under ambient conditions, the relative position of the metallic tip with respect to the excitation laser spot can shift in the lateral direction. This causes degradation of optical fields at the tip apex, resulting in the deterioration of the plasmonically enhanced Raman signal of the sample (left panel in Fig. 1A). In addition to the drift in the lateral direction, a vertical drift of the laser focus along the optical axis also occurs during the measurement over an extended period of time, which causes instability of the focal plane over the time, resulting in the decrease of scattered optical signal (right panel in Fig. 1A). Because of these uncontrollable drifts, conventional optical nanoimaging by means of an AFM-based TERS system must be completed typically within 30 min; otherwise, optical signal deteriorates beyond the acceptable level. The restriction of measurement time prevents one from nanoimaging large-sized samples to investigate the distribution of their physical, chemical, and structural properties or catalytic active sites at the nanoscale.

Because TERS imaging involves scanning the sample and measuring Raman spectra at the step of a few nanometers, it is practically very challenging to maintain the entire measurement setup stable at a nanometer scale for the entire measurement period. Raman scattering is known to be a weak process, and even with plasmonic enhancement of several orders of magnitude, the intensity of Raman scattered light is not measurable unless collected at least for a few hundred of milliseconds. This means, for example, to scan a sample of the size of 1 μm^2^ at the step of 10 nm, one needs to perform TERS measurement for at least about 30 min. This is almost the limit where the stability of the measurement system can be practically maintained under ambient conditions. Therefore, it is impossible to perform AFM-based TERS imaging on a sample that is a few micrometers in size, because such a measurement would need much longer time. This is the reason why there are no reports on TERS imaging of larger samples at nanoscale spatial resolution. However, this is very much a demand of the time to perform such a long-duration TERS imaging to study active materials used in, for example, optoelectronic devices, because they are usually a few micrometers in size, and it is a must to characterize the entire sample for its chemical composition, defects, or other irregularities that may remain localized within a few nanometers but ultimately affect the device performance. TEPL imaging of monolayer 2D materials and organic photovoltaic devices has been demonstrated for understanding their optoelectronic behaviors, where large areas of the samples ranging from a few- to hundred-micrometer scale were imaged with nanoscale spatial resolution (*7*–*9*). However, this is the case only when one measures photoluminescence or fluorescence, which involves much efficient processes than Raman scattering. There are, of course, other samples also, such as biological samples, which require nanoscale optical imaging of large areas.

Here, we demonstrate optical nanoimaging of a micrometer-sized 2D material by means of AFM-based TERS for 6 hours, which is 12 times longer than conventional nanoscale imaging period. For this purpose, we developed ultrastable TERS setup that has a homebuilt feedback system to compensate possible drift in all three dimensions (Fig. 1B). This technical development overcomes the long-standing issue of the system drift, and thus the imaging time is no longer limited by the mechanical drift.

Tungsten disulfide (WS_2_) belongs to the family of transition metal dichalcogenides (TMDCs), which are layered materials, made essentially from stacks of atomically thin layers in the bulk form. They have strong intralayer covalent bonds between the atoms, and the neighboring layers are linked to each other by very weak van der Waals interlayer interactions. The weak interlayer interaction makes it easier to exfoliate the bulk into monolayered or a few layered 2D sheets (*29*). A single layer of WS_2_ consists of a hexagonal sheet of tungsten atoms sandwiched between two hexagonal planes of sulfur atoms, which are all covalently bonded to each other to form a trilayer in a trigonal prismatic coordination. What makes a 2D TMDC much more attractive than its well-known competing material in the 2D family, graphene, is the existence of a bandgap (*30*). The absence of a bandgap in monolayered graphene limits its applications in optoelectronic devices, whereas other materials in the 2D family, namely, from black phosphorus to TMDCs to hexagonal boron nitride, have nonzero bandgaps in the increasing order from narrow to wide bandgap, with TMDCs right in the middle with versatile applications. This material also finds its frequent application in photodetectors (*31*), transistors (*32*), or photovoltaic devices (*33*). Furthermore, WS_2_ is known to have a strong spin orbit coupling and splitting that makes it an appealing material for its application in spintronics (*34*). There have been substantial efforts to realize these applications; however, performance of the large-area active 2D materials often falls below theoretical expectations because of structural defects (*35*, *36*). Almost all 2D materials inevitably have several kinds of structural defects, such as nanoscale defects, grain boundaries, and the irregularities due to step edges of layers, which can seriously affect their optical and electronic properties, resulting in degraded device performance. These nano- and microscale heterogeneities are indeed randomly distributed on the surface of 2D materials in large area, and therefore, a quantitative evaluation of the distribution of these defects in a large area of the sample ranging over several micrometers would be demanded for accelerating device performance. Because of its versatile applications and the need for a nanoscale technique to investigate large-sized samples, a monolayer and a few layers of WS_2_ is a great candidate to investigate in our attempt to showcase the strong capabilities of our technical development of TERS microscopy, where we demonstrate long-time TERS imaging without any deterioration of optical signal.

Our newly developed ultrastable optical nanoimaging system enables characterization of nanoscale defects in large-sized WS_2_ layers at a high pixel resolution down to 10 nm without losing substantial optical signal. Because our TERS system allows nanoscale spectroscopic investigation of WS_2_ layers for several-micrometer scale in size, which is impossible to perform with a conventional TERS system, we could reveal that the defect density on the surface of WS_2_ layers in large area equivalent to the device scale was indeed higher than the previously reported defect density of the smaller area. Furthermore, such long-duration TERS imaging led us to find rare properties of the materials, such as unique defects of WS_2_, which one can easily miss with conventional TERS systems. The present work paves the way for nanoscale optical spectroscopy and imaging of large-sized sample not only for optoelectronic devices but also biological and polymer samples that require relatively large-area investigation or long exposure time for obtaining sufficient optical signal.

## RESULTS

### Real-time focus stabilization with nanometer precision

**Fig. 2.**
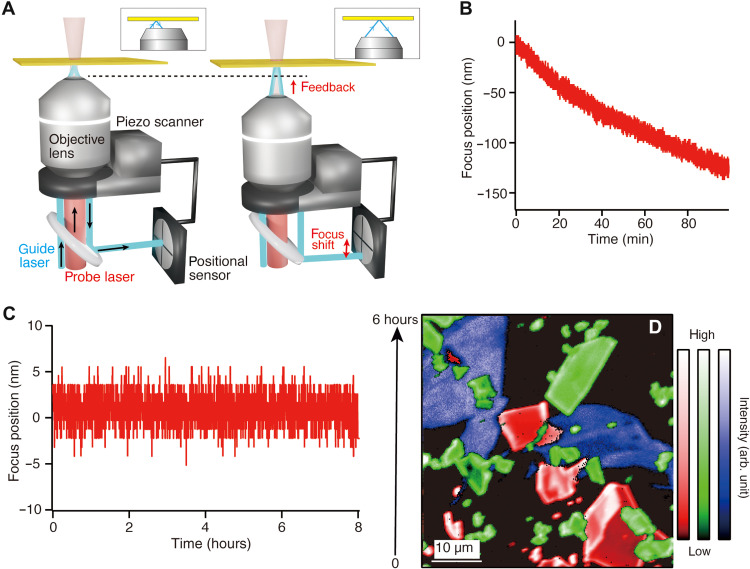
Focus drift compensation system. (**A**) Simplified illustration of the optical system for focus drift correction using laser reflection–based positional sensing (left) when the incident laser focuses on the sample plane and (right) when the incident laser is defocused. The insets show the optical path of reflected light with focusing and defocusing. (**B** and **C**) Displacement of time-dependent focus position along the optical axis (B) without feedback and (C) with feedback. (**D**) Long-time far-field Raman imaging of graphene, MoS_2_, and WS_2_ flakes transferred on a coverslip one by one. The distribution of graphene, MoS_2_, and WS_2_ flakes is visualized by blue, green, and red colors, respectively.

Over the years, a number of focus drift compensation systems have been developed for long-time optical imaging; however, it is hard to combine them with the tip drift correction method and apply into near-field optical measurement because of complexity of systems and low precision to maintain the focal plane of incident light (*37*, *38*). Therefore, we developed a focus drift correction method optimized for near-field optical measurement by means of a piezo-controlled objective scanner and laser reflection sensing scheme. Our method only requires a guide laser and a lateral positional detector to sense the displacement of the laser focus, which is applicable to a wide range of optical systems. Figure 2A depicts a schematic illustration of our focus stabilization system. A piezo-controlled objective scanner (SFS-D00100, nanoFaktur) was coupled with the objective lens to stabilize the laser focus position along the optical axis. A guide laser with a wavelength of 488 nm (Sapphire SF NX 488, Coherent) was introduced into the optical microscope with a high incident angle. The reflected light was detected by a lateral position sensor (PDP90A, Thorlabs). The lateral position of the reflected light at the sensor is associated with the displacement of the focus plane in the optical axis, as represented by the insets in Fig. 2A. Therefore, the detected positional signal travels through a closed-loop piezo controller to provide a feedback signal to the objective scanner, which maintains the focus plane. First, the accuracy and the stability of our focus drift correction system were evaluated to ensure the capability of long-time near-field optical imaging. The positional signal of the focus shift was measured and converted into positional information of the displacement in the position of the focus plane by means of calibration curves. Figure 2 (B and C) shows the displacements in the position of the focus plane measured for 100 min without feedback and for 8 hours with feedback, respectively. The focus position was not stabilized and shifted by a few hundred nanometers, while the feedback system was OFF as shown in Fig. 2B. Note that the optical signal almost vanished after 100 min when no feedback was used. In contrast, the focus position was tightly fixed within ±5 nm, while the feedback system was ON as shown in Fig. 2C. The minor fluctuation of the focal plane was attributed to possible vibrations of the optical table or the microscope and electrical noise. It was confirmed that the 5-nm fluctuation of the focus position did not significantly affect tip-enhanced Raman signal and scattering imaging of the metallic tip, as shown in fig. S1. We then demonstrated long-time confocal far-field Raman imaging of a 2D material sample, where molybdenum disulfide (MoS_2_), WS_2_, and graphene flakes coexisted on a cleaned coverslip by using our focus drift correction system. Figure 2D shows a large-area far-field confocal Raman image of the sample as an example, which was constructed from superposition of three different Raman intensity images of graphene (blue, G band at 1590 cm^−1^), MoS_2_ (green, *A*_2g_ mode at 643 cm^−1^), and WS_2_ (red, *A*_1g_ mode at 422 cm^−1^). The image size was 50 μm by 50 μm, the step size was 195 nm per pixel, and the exposure time was 0.33 s per pixel, which took about 6 hours to acquire the whole image. The far-field Raman image, without any degradation of the contrast of the image, proved that the focus position was tightly locked during the measurement. Without feedback of the objective, far-field Raman image could be measured for approximately 1 hour, after which the scattered signal degraded (*39*). Note that, because this is a far-field image, a possible nanometric instability in the lateral direction does not affect the image significantly. Our focus stabilization system has a wide range of compatibility on objective lenses, including oil or water immersion and dry objective lens. Because our focus drift correction method is based on acquisition of the reflected light, sampling rate can be of the order of millisecond, a much better value than other focus compensation systems adjusting focus position based on image capture, which, unlike our system, severely limit the temporal resolution of focus compensation.

### Laser scanning–assisted tip drift compensation

**Fig. 3.**
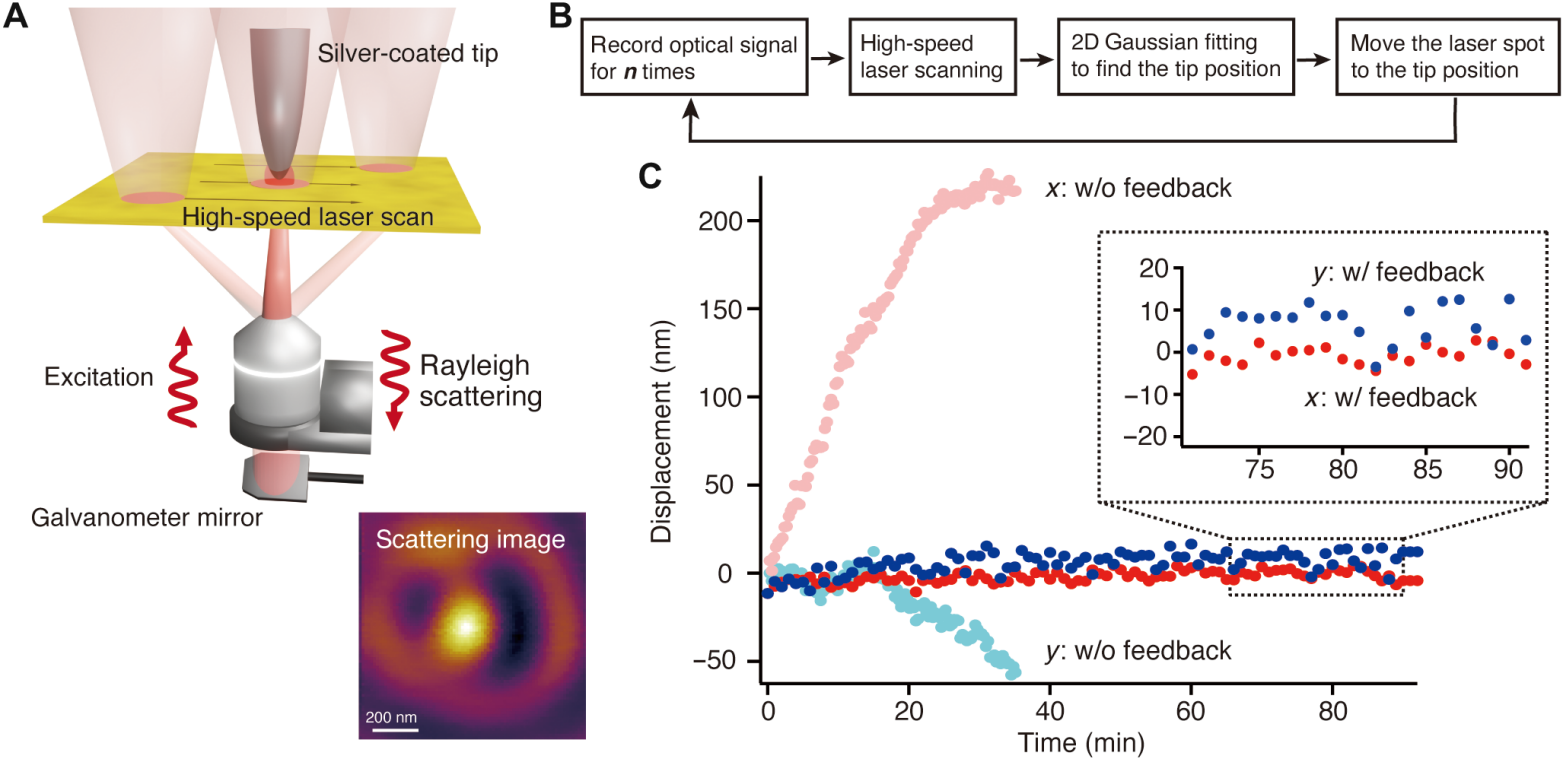
Laser scanning–assisted tip drift compensation system. (**A**) Schematic illustration of laser scanning–assisted tip drift compensation technique. Galvanometer mirrors enable to quickly scan the laser spot around the tip and to obtain a Rayleigh scattering image. (**B**) Flowchart of tip drift compensation during optical measurement. (**C**) Time-dependent lateral displacement of the relative position between the tip and the laser spot. Solid circles with red and blue color show the displacement with feedback, and solid circles with light red and light blue color show the displacement without feedback. Inset shows a zoomed graph of displacement of the relative position showing that localization precision was less than 10 nm.

After compensating for the focus drift, we proceeded to establish a tip drift compensation mechanism. A variety of solutions for tip drift have been developed, such as atom tracking technique (*40*), interferometric detection (*41*), and scattering detection (*42*), but they are not suitable for combining with the TERS system because of restriction of working environment and samples. We therefore developed our own drift compensation system based on a laser scanning–assisted technique reported previously (*43*). Figure 3A shows the concept of our laser scanning–assisted tip drift compensation. Galvano scanners were used to raster scan the incident laser spot at high speed around the metallic tip as it approached the surface of the substrate to obtain a scattering image of the metallic tip. In the scattering image, a single bright spot should appear, which originates from the strong scattering from the apex of the metallic tip because of the plasmonically excited longitudinal electric field at the tip apex. The single bright spot thus indicates the position of the metallic tip. Our lateral drift correction system takes the advantages of quick scattering imaging. Figure 3B shows a flowchart of the feedback system for tip drift compensation and optical measurement. Optical measurement begins, and optical signal from a sample is collected for ***n***th pixel, where one can arbitrarily set ***n***. Subsequently, the laser spot is scanned around the metallic tip to acquire a scattering image. The scattering image is processed with a 2D Gaussian fitting algorithm to precisely determine the center coordinate of the bright spot and hence to calculate the displacement of the tip. The relative position between the metallic tip and the laser spot was accordingly corrected, which is compensated by the displacement. After the laser scanning–assisted tip drift compensation is completed, collection of optical signal from the sample is restarted. Through the automatic processes described above, which takes only 1 s, the relative position between the tip and the laser focus can be automatically and quickly compensated during optical measurements without notable time loss. We started by evaluating the performance of the lateral tip drift correction system. We monitored the time-dependent displacement of the relative position between the tip and the laser spot every 30 s for 35 min without feedback and for 90 min with feedback. In Fig. 3C, the tip was initially located at the center of the laser focus at 0 s. The relative displacement became larger over the time, and lastly, the tip drifted by more than 200 nm in the *x* direction, while it drifted by 60 nm in the *y* direction, which means that the tip was clearly out of the center of the focus spot in 35 min without feedback. We would like to note here that the measurement without tip drift compensation could not be performed for more than 35 min, because the displacement of the relative position was out of the working range of our system to track the relative position. In contrast, the lateral tip drift correction system allows the tip to remain localized within ±10 nm from the center of the focus spot for 90 min in Fig. 3C. It should be noted that the localization precision of the relative position between the tip and the laser spot depends on time interval between drift correction process, which was examined, as shown in fig. S2.

### Stabilization of near-field optical signal

**Fig. 4.**
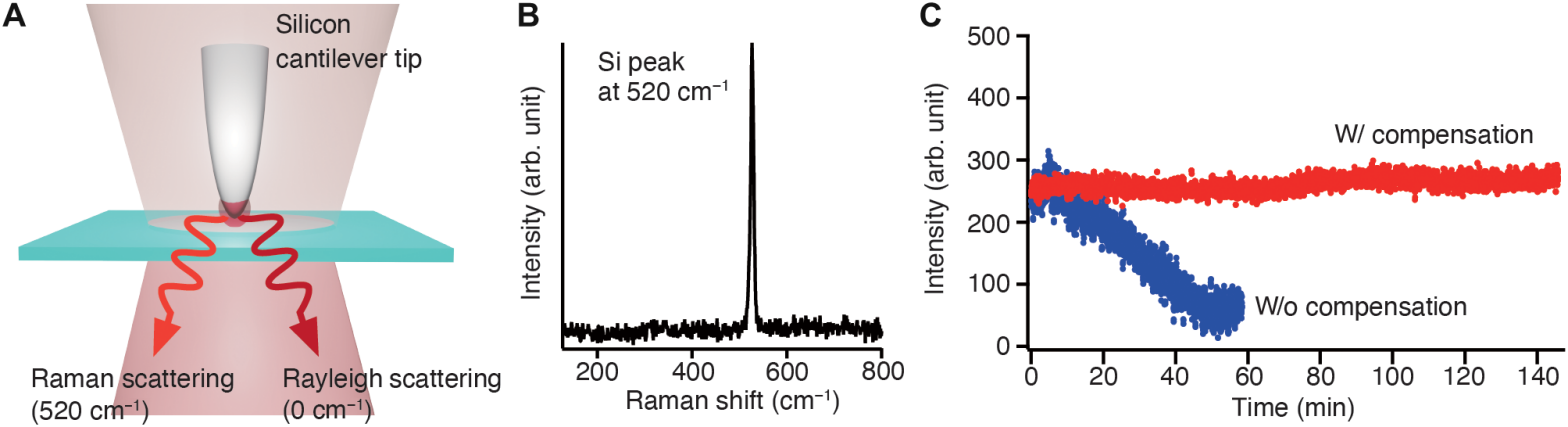
Demonstration of long-duration near-field optical measurement. (**A**) Schematic of nano-optical measurement employing Raman scattering signal of a silicon nanotip. (**B**) Representative Raman spectrum of a silicon tip when the silicon tip is approached to the glass surface. LO phone mode of silicon appears at 520 cm^−1^. (**C**) Time-dependent Raman intensity of silicon peak at 520 cm^−1^ was measured for 150 min with 3D drift correction system and for 60 min without any drift compensation.

To ensure that optical signal is not degraded during tip-enhanced optical measurements owing to the stabilization of the focus position along the optical axis and the relative position between the tip and the laser spot in the lateral direction, we measured time-dependent intensity of Raman scattering signal of a silicon tip. When a bare silicon tip is approached on a glass substrate and brought into the center of an incident focus spot, Raman scattering of silicon can be obtained from the nanometric volume of the tip apex, as shown in Fig. 4A. Because the intensity of Raman signal of silicon is dependent on the relative position between the tip and the laser spot along both the optical and the lateral axes and a silicon tip is not photodegraded, the Raman signal of silicon can be used as a marker Raman signal to confirm stabilization of near-field optical signal (*42*, *44*). Figure 4B shows a typical Raman spectrum of a silicon nanotip approached to a glass substrate, where the longitudinal optical (LO) phonon mode of silicon at 520 cm^−1^ can be observed. Figure 4C shows time-dependent Raman signal intensity of the silicon LO phonon, measured with 3D drift compensation for 150 min and without drift compensation for 30 min. Raman signal of silicon shows highly stable intensity for 150 min when drift compensation system was on without any notable signal loss, while it reduced to almost zero after 30 min when drift compensation was off. This indicates that the developed drift compensation system enables long-duration near-field optical measurement in a stable manner.

### Long-time tip-enhanced optical nanoimaging of WS_2_

**Fig. 5.**
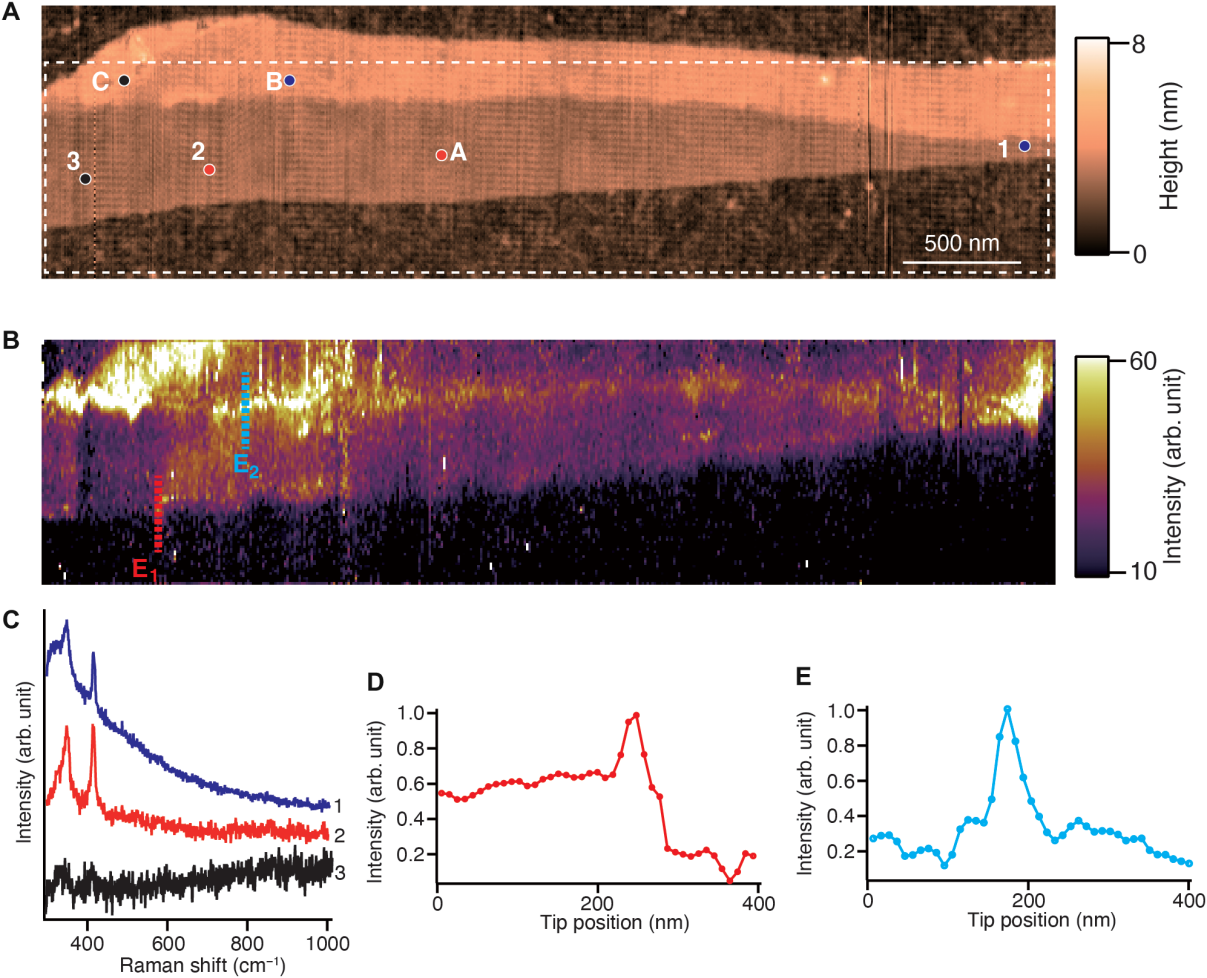
Long-time TERS imaging of WS_2_ layers. (**A**) Height image of large-sized WS_2_ layers. (**B**) TERS image of the corresponding area of the height image, reconstructed by the intensity of the *A*_1g_ mode. (**C**) TERS spectra of the WS_2_ layers measured at areas 1 to 3 marked in the TERS image. (**D** and **E**) Line profiles of TERS intensity at the *A*_1g_ mode along the dotted lines of (D) E_1_ and (E) E_2_ shown in the TERS image.

We then proceeded to demonstrate long-time, high-resolution optical nanoimaging of large-sized WS_2_ layers by our stabilized TERS system. WS_2_ can be identified by its distinctive Raman modes of *E*^1^_2g_ and *A*_1g_ as shown in the Raman spectrum of bulk WS_2_ in fig. S3A. The *E*_2g_ mode arises from the in-plane vibration of sulfur and tungsten atoms, while the *A*_1g_ mode originates from the out-of-plane oscillation of sulfur atoms. For TERS imaging, we have used silver-coated tips because they provided high signal enhancement. Figure 5A shows an AFM height image of a large few-layered flake of WS_2_. The variation of the thickness of WS_2_ layers can be visualized by the topographic contrast. Figure 5B shows a TERS intensity image of the corresponding area, constructed by the intensity of *A*_1g_ mode of WS_2_. The area for TERS imaging is marked by the dashed line in Fig. 5A. The TERS image is slightly shifted with respect to the AFM image, because the AFM and TERS image were measured separately; however, it does not affect our analysis of the TERS image here. The scan area was 1 μm by 4 μm, which is at least four times larger than typical TERS images of 2D materials recorded by conventional AFM-based TERS systems, while the step size was set to 10 nm, which is similar or smaller than sizes used in typical TERS and TEPL images of 2D materials (*45*, *46*). It should be noted that optical nanoimaging of 2D materials in relatively large area may be performed by either significantly increasing the measurement step size to more than 30 nm, which will deteriorate the spatial resolution, or by using relatively high excitation power. However, because WS_2_ easily undergoes photo-oxidation with visible light (*47*), the low incident power of less than 40 μW must be used to avoid photodegradation of the WS_2_ sample, thereby the exposure time was set to be 0.54 s per pixel in our experiment. Because the critical length of the spatial resolution probing the heterogeneities on 2D materials is typically less than 30 nm (*18*), the measurement step size also must be smaller than 30 nm. Hyperspectral nanoimaging in Fig. 5B was carried out for 6 hours, which is 12 times longer than conventional optical nanoimaging. Some variation of TERS intensity was observed in the TERS image, which suggests that heterogeneities are randomly distributed in the wide scale. We observed the variation of TERS signal intensity depending on the number of WS_2_ layers in the areas marked as A to C in the height image. The areas A to C were identified as two- to four-layered regions of WS_2_, respectively, as probed by the frequency shift of the *A*_1g_ mode of WS_2_, as indicated by the dotted line in fig. S3B in the Supplementary Materials. We would like to mention here that the gap-mode TERS intensity of WS_2_ is not always proportional to the number of layers, because the resonance condition of the plasmonic gap between the metallic tip and the metallic substrate and the electronic energy band of WS_2_ are modified when the number of layers varies. We further consider locations 1 to 3 within the two-layered area A, as marked in Fig. 5A. We observed a high TERS signal around the location 1 in Fig. 5A, compared to other basal areas, although the number of layers was the same. TERS spectra recorded at locations 1 and 2, which is assumed as a basal plane and bilayered WS_2_, are shown in Fig. 5C, where a strong photoluminescence signal accompanied the TERS signal of WS_2_. This is due to the local change of surface charge conditions of WS_2_, resulting in resonant excitation of Raman scattering and photoluminescence of WS_2_, reported in a previous work (*20*). Sample area around location 3 is categorized as a grain boundary area generated during the exfoliation process, as proved by the disappearance of TERS intensity of WS_2_ shown in Fig. 5C. Such physical irregularities at the atomic level perturb natural vibrations of atomic bonds, so that Raman signal intensity associated with defects reduces in comparison with that of a perfect lattice. The reduction in both Raman signal and photoluminescence intensity by defects is indeed consistent with the results reported previously (*16*, *18*). The other type of defects is the step edges of WS_2_ layers that were visualized by the rise of TERS intensity in the TERS image. The continuous step edges between two WS_2_ layers along the length scale of a few micrometers were visualized in the TERS image. The sudden increase of TERS signal intensity at the step edges between two WS_2_ layers as well as between a WS_2_ layer and the substrate were observed as shown in Fig. 5 (D and E), which was confirmed by the line profiles along the cyan lines E_1_ and E_2_, respectively, marked in Fig. 5B.

**Fig. 6.**
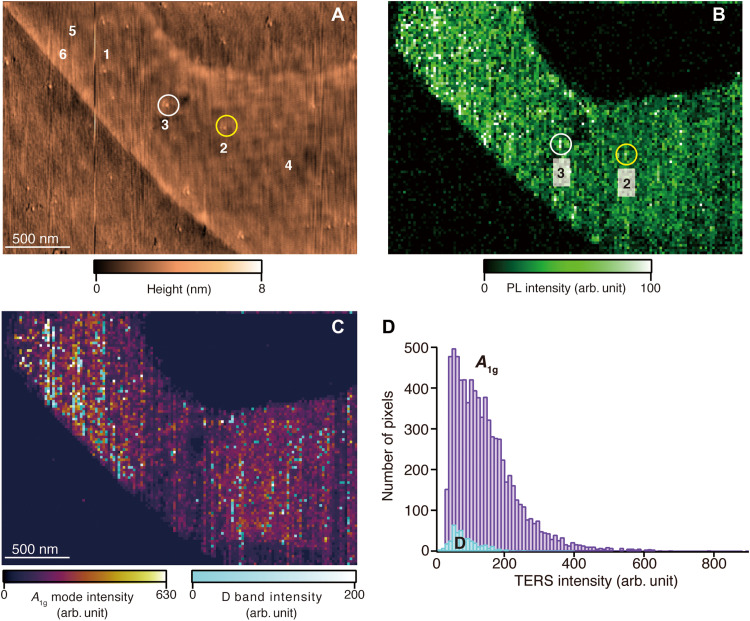
Nanoscale defect analysis of large-sized WS_2_. (**A**) AFM image of a large-area WS_2_ monolayer. (**B**) TEPL image of the corresponding area. The yellow and white circles shown in both images represent the nanoscale protrusions that enhance photoluminescence (PL) of the WS_2_ monolayer. (**C**) Superposition of the TERS intensity image of the *A*_1g_ mode and the D band. (**D**) A histogram of the intensity of *A*_1g_ mode (purple) and the D band (light blue) in 7731 and 402 points, showing the defect density of 5.2% in our WS_2_ sample.

We further applied our nanoimaging system for the evaluation of the spatial distribution of other types of defects, such as nanoscale protrusions and sulfur vacancies for a large-sized WS_2_ monolayer. Sulfur vacancies in 2D materials can significantly degrade their optoelectronic properties by introducing a new intermediate state that engenders anomalous energy upconversion (*48*). Figure 6A shows an AFM height image of a large-sized WS_2_ monolayer. In the AFM image, several nanoscale protrusions on the WS_2_ monolayer are observed. Typical protrusions are shown by yellow and white circles and are also marked as regions 2 and 3 in the AFM image. It can be speculated that such nanoscale protrusions can be attributed to either the formation of few nanometer-sized tungsten oxide, leading the topographic change of photo-oxidized WS_2_ layer, or small grains originating from the metallic substrate. Figure 6B shows a TEPL image of the corresponding area, where the TEPL signal intensity visualizes the structure of WS_2_. In Fig. 6B, strong TEPL signal was observed from the nanoscale protrusions, as shown by regions 2 and 3. TERS spectra recorded from regions 2 and 3 are also shown in fig. S4A, together with the TERS spectrum of basal plane of WS_2_ marked as region 1 in the AFM image. The far-field Raman spectrum recorded from the same sample was also shown in fig. S4A to estimate the TERS contrast and enhancement factor. We found that the protrusions yielded drastic enhancement of both TERS and TEPL signal of WS_2_. Because a photo-oxidized WS_2_ layer usually shows the decrease of TERS and TEPL signal intensity, the present result led us to conclude that the nanoscale protrusions are small metallic grains that are formed during fabrication process of the metallic substrate. The increase of TERS and TEPL signal intensity can be attributed to either that the energy bandgap of WS_2_ is modified and gets closer to the wavelength of the excitation laser because of local strains generated by the metal grains (*26*) or the fact that these nanoscale metal grains can locally and strongly confine light due to the lightning rod effect, which would result in giant field enhancement.

We also evaluated defects originating from sulfur vacancies by observing the D band scattering of WS_2_ (*49*). Figure S4B shows TERS spectra extracted from two different positions, marked as 4 and 5 in Fig. 6A. In the TERS spectra recorded at area 4, only vibrational modes of WS_2_, such as *E*^1^_2g_ (~350 cm^−1^) and *A*_1g_ (~420 cm^−1^) modes are observed, which indicates that area 4 is the basal plane of WS_2_ without defects. The TERS spectra at area 5 shows that not only those primary vibrational modes but also a distinctive peak at the left-hand side of the *A*_1g_ mode, called the D band (410 cm^−1^), can be observed. The D band indicates the existence of sulfur vacancies in the WS_2_ monolayer. Another defect-related scattering, called the D′ band (433 cm^−1^), was also observed at some locations, as shown in fig. S4C. The D′ band is an infrared-active mode and originally weak; hence, it was not observed at all areas, where D band was observed. Figure 6C shows a TERS intensity image (*A*_1g_ mode) of a large-sized WS_2_ monolayer, which is superimposed with the D band intensity image. The TERS image clearly identifies the positions, where sulfur vacancies are located on the surface. This is the advantage of high-resolution TERS imaging, providing spectroscopic evidence of such surface features, which cannot be identified by the conventional AFM image shown in Fig. 6A. Figure 6D shows a histogram of TERS intensities of *A*_1g_ mode (purple) and D band (light blue) obtained from 7731 and 402 pixels, respectively, taken from Fig. 6C. Subsequently, we calculated a density (*D*) of nanoscale defects in the WS_2_ monolayer using the total pixel numbers of *A*_1g_ mode (*N*_A_) and D band (*N*_D_) to quantitatively evaluate defect areas in the large-sized WS_2_ monolayer. The density was calculated by the following equation

$$D\;(\%)=\frac{N_{D}}{N_{A}}\times 100$$

In our WS_2_ sample, the density of nanoscale defects was estimated to be 5.2%, while in previous studies on evaluation of nanoscale defects in small-sized 2D materials (*36*, *50*), the defect density was reported to be 1 to 3%. We would like to mention here that our defect analysis has been conducted for an area larger than 4,000,000 nm^2^, keeping the spatial resolution to nanoscale, similar to previous studies based on high-resolution optical nanoimaging. Our results indicate that nanoscale defects existed in 2D materials with a certain density in the large scale that is equivalent to the device scale and is indeed higher than the defect density previously investigated for the smaller area. Furthermore, our system also enabled to observe rare events or properties of a sample, such as D′ band of WS_2_ layers. In our WS_2_ sample, D′ band was observed only at 28 pixels in the TERS image, which is only 0.2% of the whole imaging area. Such minor properties of the sample could be easily missed to be detected when a smaller area of the sample was imaged. Last, we would like to mention here that there was no notable loss of TERS signal of WS_2_ layers during whole imaging. Figure S5 (A and B) shows five TERS spectra obtained from the first few line scans and from the last few line scans during the TERS imaging of WS_2_ layers shown in Fig. 6C. We have also plotted the averaged values and the SDs of TERS intensity (*A*_1g_ mode) obtained from those five TERS spectra in fig. S5C. These results clearly indicate that the TERS signal of WS_2_ layers was stable during the whole imaging process, and there was no notable degradation of TERS signal. At the most, only less than 10% average decrease of TERS signal between the first few and the last few spectra was observed, which is well within the acceptable range for constructing TERS images. We have demonstrated that our technological advancement in TERS imaging has made it possible to investigate nanoscale properties of a large-sized sample. This is extremely useful not only for WS_2_ but also for many other interesting photosensitive materials, such as HfO_2_, and 2D polymers, which are frequently used in various devices. These nanoscale defects significantly modify their optoelectronic properties, leading to deteriorated device performance. In contrast, the presence of defects indeed tailors unique properties of 2D materials, for example, engineering bandgap by inducing defects. Therefore, large-scale defect analysis is crucial for both evaluation and development of their applications. Further studies on characterizing defects and correlating them with electrical and mechanical properties of materials in large-scale would be essential for deeper understanding of their intrinsic properties and optimization of nanodevices based on 2D materials.

## DISCUSSION

In conclusion, we demonstrated hyperspectral nanoimaging of large-sized WS_2_ layers by developing the ultrastable TERS system. Our optical system compensates both tip drift and focus drift with nanometer precision, which avoids notable loss of optical signal in near-field optical measurement with time even for a long period. Owing to the high stability of our optical system, hyperspectral optical nanoimaging of micrometer-sized WS_2_ sample was achieved, revealing various randomly distributed defects. TERS imaging was carried out for 6 hours with our setup, which is 12 times longer than conventional optical nanoimaging, and the scan area of the TERS image was extended at least four times larger than typical TERS images of 2D materials recorded by conventional AFM-based TERS systems, while the step size was kept to as small as 10 nm, which provided a spatial resolution high enough to resolve heterogeneities of WS_2_ layers. It should be noted that, although we have demonstrated long-duration TERS imaging for up to 6 hours in this study, our ultrastable TERS system has overcome a crucial drift issue and thus has, in principle, no limitation of imaging time, unless other possible factors limit it such as tip degradation due to oxidation. Kumar *et al*. (*51*) demonstrated that the plasmonic enhancement of silver-coated AFM-TERS probes rapidly degrades in ambient environment because of surface oxidation. However, as our results confirm in Figs. 5 and 6, our careful control over the humidity, temperature, air purity, and the enclosure of the experimental setup was good enough to keep the silver tip with an acceptable level of degradation for 6 to 7 hours. A careful control of the ambient condition is extremely important if one uses silver as the plasmonic material and wants to use it for a long period of several hours.

Another possible reason why the plasmonic enhancement of our silver-coated tips could be well maintained for several hours is the fact that we used the gap-mode configuration supported by a gold thin substrate, which is stable under ambient conditions. It is known that the nanogap between a metallic nanotip and a metallic substrate plays a crucial role in total plasmonic enhancement, the strength of which is weakly dependent on plasmon resonance of the tip alone. While plasmon resonance of a tip alone can be significantly affected by the surface morphology and surface conditions of the tip, the gap-mode enhancement is only weakly dependent on the same. Therefore, when a silver tip is used without a metallic substrate, the total enhancement degrades much faster compared to the case when it is used with a metallic substrate. We believe that this could have also added to the lifetime of the stable enhancement in our experiments. One of the possible ways to protect plasmonic enhancement of silver-coated tips is the use of dielectric protective layers for silver tips as demonstrated in previous studies (*22*, *52*).

Long-time TERS imaging enabled us to observed rare properties and events of materials, which could be usually missed by imaging a smaller area. This would lead to uncover novel properties and vast heterogeneities of various samples through TERS imaging, resulting in discovery of new subpopulations and features of samples. Hence, our stabilized optical nanoimaging system would open paths for numerous nanoimaging applications in various fields; for example, surface analysis of optoelectronic devices using large-sized 2D materials, such as graphene and TMDCs, in which local heterogeneous nanostructures coexist in the large scale, and structural elucidation of 2D polymer monolayer, where the polymerization conversion rate of synthesized polymers has to be evaluated in a large area of several micrometers. Although quantitative analysis of defect densities has been performed by using a high-resolution electron microscopy (*36*, *53*), optical nanoimaging enables not only to detect heterogeneities but also to spectroscopically characterize defects.

Other important applications that could be benefited from our technique are nano-optical imaging with long acquisition time of Raman scattering signal or with extremely small step size. For example, some biological samples, such as proteins and lipid bilayers, have extremely weak cross section of Raman scattering (*54*–*56*), thereby long acquisition time of typically several seconds is required to obtain a TERS spectrum. Low-frequency Raman vibrational imaging of 2D materials, which allows one to characterize interlayer defects and local stacking configurations at nanoscale, also requires long acquisition time, because the interlayer vibrational modes are originally weak (*57*). In addition, our system can also be applied into single-molecule imaging with high pixel resolution, which has recently attracted increasing attentions in the field of TERS and TEPL. Single-molecule TERS imaging with the subnanometer spatial resolution offers the capability for fundamental investigation of the vibrational modes (*13*). In such extreme imaging conditions, the step size of TERS imaging has to be set to the subnanometer scale; otherwise, vibrational modes of individual molecules cannot be resolved. It therefore only enables an isolated molecule to be imaged. Intramolecular-resolution TERS imaging with high pixel numbers would help to study the vibrational and chemical properties of multiple molecules, which would allow to obtain detailed information of molecules on the surface. This would be possible only if measurement is stable for a long time. The achievement obtained in this work would lead further applications of nano-optical imaging using AFM-based TERS and unveil interesting and remarkable physical, optical, and chemical properties of nanostructured materials.

## MATERIALS AND METHODS

### Sample preparation

Ultrasmooth gold thin films were used for gap-mode TERS measurement (*58*). Au (99.95%; Nilaco) was thermally evaporated onto a piranha-cleaned glass coverslip followed after precoating a 2-nm Cu buffer layer in a vacuum chamber to form a smooth layer of gold with a thickness of 10 nm. By optimizing the evaporation parameters and having a Cu buffer layer (*59*), the surface of the gold thin film was extremely smooth. We thus believe that the smooth surface of the gold thin film did not have any hot spots on the surface and hence did not bring about surface-enhanced Raman signal by the substrate.

### Preparation of monolayer WS_2_ sample

A large-sized WS_2_ monolayer and a few-layer samples were fabricated by the gold-assisted mechanical exfoliation method (*29*). First, a fresh surface of bulk crystal of WS_2_ was cleaved by the blue tape (Nitto) and was gently put onto the gold thin film. After pressing the tape vertically for about 5 to 10 s, the tape was removed from the substrate, so that large-sized monolayer and a few layer flakes could be easily obtained.

### Metallic nanotip for TERS

TERS tips were prepared by thermal evaporation of an 80-nm-thick silver layer onto oxidized silicon cantilevers (CSG01/Bare, NT-MDT) at a rate of 0.4 Å/s. A scanning electron microscope image of the apex of a typical silver-coated granular tip is shown in fig. S6.

### TERS setup

The schematic illustration of our TERS setup is shown in fig. S7. All TERS measurements were performed on a bottom-illumination TERS setup that combines an AFM system (MFP-3D-Bio, Oxford Instruments) with an inverted optical microscope (ECLIPSE Ti-U, Nikon) coupled with a single spectrometer (Acton SP-2300, Princeton Instruments) and a Peltier-cooled charge-coupled device camera (PIXIS 100, Princeton Instruments). The spectrometer disperses light through a grating with a period of 1200 grooves per millimeter. A radially polarized 638-nm laser (DL640-025-S, CrystaLaser), which is the incident light source for TERS measurement, was created by polarization elements including a Z-pol (Nanophoton). An oil immersion objective [100×, numerical aperture (NA) 1.45] purchased from Olympus was used for both excitation and collection of the TERS signals. A mask was used to block the low-NA component of the incident beam. The backscattered signals were filtered with a long-pass filter, and only Raman scattered light was guided into the detection system. The laser scanning system has a pair of galvanometer mirrors (GVS002, Thorlabs). The rotational axes were orthogonal to each other, enabling the laser beam to raster scan in lateral direction on the substrate. The galvanometer mirrors were placed at the optical plane conjugate to the entrance pupil of the objective lens by way of relay optics, so that the incident laser can be scanned without modification of optical alignment while scanning the mirrors. Scattering light from the metallic tip was detected by an auto-balanced silicon photodiode (PDA36A2, Thorlabs), which provides us a scattering image of the metallic tip. An 860-nm super luminescent diode light was used for the deflection of the AFM cantilever, which was later completely cut out from the optical path to the silicon photodiode by a short-pass filter. The optical microscope was enclosed by a homemade enclosure to minimize thermal drift of AFM.

The contact mode configuration of AFM system was used for both topographic imaging and TERS measurement in this work. TERS data were analyzed by a homemade IGOR program.

## References

[1] X. Wang, K. Broch, F. Schreiber, A. J. Meixner, D. Zhang, Revealing nanoscale optical properties and morphology in perfluoropentacene films by confocal and tip-enhanced near-field optical microscopy and spectroscopy. Phys. Chem. Chem. Phys. 18, 15919–15926 (2016). 27241888 10.1039/c6cp01153e

[2] R. M. Stöckle, Y. D. Suh, V. Deckert, R. Zenobi, Nanoscale chemical analysis by tip-enhanced Raman spectroscopy. Chem. Phys. Lett. 318, 131–136 (2000).

[3] B. Pettinger, G. Picardi, R. Schster, G. Ertl, Surface enhanced Raman spectroscopy: Towards single molecule spectroscopy. Electrochemistry 68, 942–949 (2000).

[4] H. Yin, L. Zheng, W. Fang, Y. Lai, N. Porenta, G. Goubert, H. Zhang, H. Su, B. Ren, J. O. Richardson, J. Li, R. Zenobi, Nanometre-scale spectroscopic visualization of catalytic sites during a hydrogenation reaction on a Pd/Au bimetallic catalyst. Nat. Catal. 3, 834–842 (2020).

[5] H.-S. Su, H.-S. Feng, Q.-Q. Zhao, X.-G. Zhang, J.-J. Sun, Y. He, S.-C. Huang, T.-X. Huang, J.-H. Zhong, D.-Y. Wu, B. Ren, Probing the local generation and diffusion of active oxygen species on a Pd/Au bimetallic surface by tip-enhanced raman spectroscopy. J. Am. Chem. Soc. 142, 1341–1347 (2020). 31893500 10.1021/jacs.9b10512

[6] G. Goubert, X. Chen, S. Jiang, R. P. Van Duyne, In situ electrochemical tip-enhanced raman spectroscopy with a chemically modified tip. J. Phys. Chem. Lett. 9, 3825–3828 (2018). 29945445 10.1021/acs.jpclett.8b01635

[7] W. Su, N. Kumar, S. Mignuzzi, J. Crain, D. Roy, Nanoscale mapping of excitonic processes in single-layer MoS_2_ using tip-enhanced photoluminescence microscopy. Nanoscale 8, 10564–10569 (2016). 27152366 10.1039/c5nr07378b

[8] A. A. Y. Guilbert, F. A. Castro, B. C. Schroeder, W. Su, I. McCulloch, N. Kumar, A. Z.-Lemanczyk, J. Nelson, D. Roy, T. Kirchartz, S. M. Tuladhar, Simultaneous topographical, electrical and optical microscopy of optoelectronic devices at the nanoscale. Nanoscale 9, 2723–2731 (2017). 28078339 10.1039/c6nr09057e

[9] W. Su, N. Kumar, H. Shu, O. Lancry, M. Chaigneau, In situvisualization of optoelectronic behavior of grain boundaries in monolayer WSe_2_ at the nanoscale. J. Phys. Chem. C 125, 26883–26891 (2021).

[10] R. Kato, S. Igarashi, T. Umakoshi, P. Verma, Tip-enhanced raman spectroscopy of multiwalled carbon nanotubes through D-band imaging: Implications for nanoscale analysis of interwall interactions. ACS Appl. Nano Mater. 3, 6001–6008 (2020).

[11] R. Kato, T. Umakoshi, P. Verma, Raman spectroscopic nanoimaging of optical fields of metal nanostructures with a chemically modified metallic tip. J. Phys. Chem. C 125, 20397–20404 (2021).

[12] T. Yano, P. Verma, Y. Saito, T. Ichimura, S. Kawata, Pressure-assisted tip-enhanced Raman imaging at a resolution of a few nanometres. Nat. Photonics 3, 473–477 (2009).

[13] B. Yang, G. Chen, A. Ghafoor, Y. Zhang, Y. Zhang, Y. Zhang, Y. Luo, J. Yang, V. Sandoghdar, J. Aizpurua, Z. Dong, J. G. Hou, Sub-nanometre resolution in single-molecule photoluminescence imaging. Nat. Photonics 14, 693–699 (2020).

[14] J. Zhong, X. Jin, L. Meng, X. Wang, H. Su, Z. Yang, C. T. Williams, B. Ren, Probing the electronic and catalytic properties of a bimetallic surface with 3 nm resolution. Nat. Nanotechnol. 12, 132–136 (2016). 27870842 10.1038/nnano.2016.241

[15] T. Huang, X. Cong, S. Wu, K. Lin, X. Yao, Y. He, J. Wu, Y. Bao, S. Huang, X. Wang, P. Tan, B. Ren, Probing the edge-related properties of atomically thin MoS_2_ at nanoscale. Nat. Commun. 10, 5544 (2019). 31804496 10.1038/s41467-019-13486-7 PMC6895227

[16] R. Kato, T. Umakoshi, R. T. Sam, P. Verma, Probing nanoscale defects and wrinkles in MoS_2_ by tip-enhanced Raman spectroscopic imaging. Appl. Phys. Lett. 114, 073105 (2019).

[17] W. Su, N. Kumar, A. Krayev, M. Chaigneau, In situ topographical chemical and electrical imaging of carboxyl graphene oxide at the nanoscale. Nat. Commun. 9, 1–7 (2018). 30038358 10.1038/s41467-018-05307-0 PMC6056528

[18] W. Bao, N. J. Borys, C. Ko, J. Suh, W. Fan, A. Thron, Y. Zhang, A. Buyanin, J. Zhang, S. Cabrini, P. D. Ashby, A. Weber-bargioni, S. Tongay, S. Aloni, D. F. Ogletree, J. Wu, M. B. Salmeron, P. J. Schuck, Visualizing nanoscale excitonic relaxation properties of disordered edges and grain boundaries in monolayer molybdenum disulfide. Nat. Commun. 6, 1–7 (2015). 10.1038/ncomms8993 PMC4557266 26269394

[19] D. Moore, K. Jo, C. Nguyen, J. Lou, C. Muratore, D. Jariwala, N. R. Glavin, Uncovering topographically hidden features in 2D MoSe_2_ with correlated potential and optical nanoprobes. npj 2D Mater. Appl. 4, 44 (2020).

[20] D. Jariwala, A. Krayev, J. Wong, A. E. Robinson, M. C. Sherrott, S. Wang, G.-Y. Liu, M. Terrones, H. A. Atwater, Nanoscale doping heterogeneity in few-layer WSe_2_ exfoliated onto noble metals revealed by correlated SPM and TERS imaging. 2D Mater. 5, 035003 (2018).

[21] A. Bhattarai, P. Z. El-khoury, Nanoscale chemical reaction imaging at the solid-liquid interface via TERS. J. Phys. Chem. Lett. 10, 2817–2822 (2019). 31074285 10.1021/acs.jpclett.9b00935

[22] N. Kumar, C. S. Wondergem, A. J. Wain, B. M. Weckhuysen, In situ nanoscale investigation of catalytic reactions in the liquid phase using zirconia-protected tip-enhanced raman spectroscopy probes. J. Phys. Chem. Lett. 10, 1669–1675 (2019). 30916970 10.1021/acs.jpclett.8b02496 PMC6477806

[23] R. B. Jaculbia, H. Imada, K. Miwa, T. Iwasa, M. Takenaka, B. Yang, E. Kazuma, N. Hayazawa, T. Taketsugu, Y. Kim, Single-molecule resonance Raman effect in a plasmonic nanocavity. Nat. Nanotechnol. 15, 105–110 (2020). 31959928 10.1038/s41565-019-0614-8

[24] X. Tian, Y. Zhang, Y. Yu, S. Jing, Y. Zhang, G.-J. Tian, Y. Luo, J.-L. Yang, Z.-C. Dong, J. G. Hou, Probing intramolecular vibronic coupling through vibronic-state imaging. Nat. Commun. 12, 1321 (2021). 33627671 10.1038/s41467-021-21571-z PMC7904785

[25] M. Kang, H. Kim, E. Oleiki, Y. Koo, H. Lee, J. Choi, T. Eom, G. Lee, Y. D. Suh, K.-D. Park, Freeze-frame approach for robust single-molecule tip-enhnaced Raman spectroscopy at room temperature. arXiv: 10.48550/arXiv.2110.12641[physics.chem-ph] (25 October 2021).

[26] T. P. Darlington, A. Krayev, V. Venkatesh, R. Saxena, J. W. Kysar, J. Nicholas, P. J. Schuck, Facile and quantitative estimation of strain in nanobubbles with arbitrary symmetry in 2D semiconductors verified using hyperspectral nano-optical imaging. J. Chem. Phys. 153, 024702 (2020). 32668931 10.1063/5.0012817

[27] A. Fali, T. Zhang, J. P. Terry, E. Kahn, K. Fujisawa, B. Kabius, S. Koirala, Y. Ghafouri, D. Zhou, W. Song, L. Yang, M. Terrones, Y. Abate, Photodegradation protection in 2D in-plane heterostructures revealed by hyperspectral nanoimaging: The role of nanointerface 2D alloys. ACS Nano 15, 2447–2457 (2021). 33464036 10.1021/acsnano.0c06148

[28] K. Braun, O. Hauler, D. Zhang, X. Wang, T. Chasse, A. J. Meixner, Probing bias-induced electron density shifts in metal-molecule interfaces via tip-enhanced Raman scattering. J. Am. Chem. Soc. 143, 1816–1821 (2021). 33492134 10.1021/jacs.0c09392

[29] Y. Huang, Y. Pan, R. Yang, L. Bao, L. Meng, H. Luo, Y. Cai, G. Liu, W. Zhao, Z. Zhou, L. Wu, Z. Zhu, M. Huang, L. Liu, L. Liu, P. Cheng, K. Wu, S. Tian, C. Gu, Y. Shi, Y. Guo, Z. G. Cheng, J. Hu, L. Zhao, G. Yang, E. Sutter, P. Sutter, Y. Wang, W. Ji, X. Zhou, H. Gao, Universal mechanical exfoliation of large-area 2D crystals. Nat. Commun. 11, 2453 (2020). 32415180 10.1038/s41467-020-16266-w PMC7228924

[30] E. Blundo, M. Felici, T. Yildirim, G. Pettinari, D. Tedeschi, A. Miriametro, B. Liu, W. Ma, Y. Lu, A. Polimeni, Evidence of the direct-to-indirect band gap transition in strained two-dimensional WS_2_, MoS_2_, and WSe_2_. Phys. Rev. Res. 2, 1–7 (2020).

[31] T. H. Tsai, Z. Y. Liang, Y. C. Lin, C. C. Wang, K. I. Lin, K. Suenaga, P. W. Chiu, Photogating WS_2_ photodetectors using embedded WSe_2_ charge puddles. ACS Nano 14, 4559–4566 (2020). 32271535 10.1021/acsnano.0c00098

[32] J. Kumar, M. A. Kuroda, M. Z. Bellus, S. J. Han, H. Y. Chiu, Full-range electrical characteristics of WS_2_ transistors. Appl. Phys. Lett. 106, 123508 (2015).

[33] Y. Zhou, H. Tan, Y. Sheng, Y. Fan, W. Xu, J. H. Warner, Utilizing interlayer excitons in bilayer WS_2_ for increased photovoltaic response in ultrathin graphene vertical cross-bar photodetecting tunneling transistors. ACS Nano 12, 4669–4677 (2018). 29671322 10.1021/acsnano.8b01263

[34] D. W. Latzke, W. Zhang, A. Suslu, T. R. Chang, H. Lin, H. T. Jeng, S. Tongay, J. Wu, A. Bansil, A. Lanzara, Electronic structure, spin-orbit coupling, and interlayer interaction in bulk MoS_2_ and WS_2_. Phys. Rev. B. 91, 1–6 (2015).

[35] Q. H. Wang, K. Kalantar-Zadeh, A. Kis, J. N. Coleman, M. S. Strano, Electronics and optoelectronics of two-dimensional transition metal dichalcogenides. Nat. Nanotechnol. 7, 699–712 (2012). 23132225 10.1038/nnano.2012.193

[36] J. Hong, Z. Hu, M. Probert, K. Li, D. Lv, X. Yang, L. Gu, N. Mao, Q. Feng, L. Xie, J. Zhang, D. Wu, Z. Zhang, C. Jin, W. Ji, X. Zhang, J. Yuan, Z. Zhang, Exploring atomic defects in molybdenum disulphide monolayers. Nat. Commun. 6, 6293 (2015). 25695374 10.1038/ncomms7293 PMC4346634

[37] X. Zhang, F. Zeng, Y. Li, Y. Qiao, Improvement in focusing accuracy of DNA sequencing microscope with multi-position laser differential confocal autofocus method. Opt. Express 26, 897–906 (2018). 29401968 10.1364/OE.26.000887

[38] K. Guo, J. Liao, Z. Bian, X. Heng, G. Zheng, InstantScope : A low-cost whole slide imaging system with instant focal plane detection. Biomed. Opt. Express 6, 3210–3216 (2015). 26417493 10.1364/BOE.6.003210 PMC4574649

[39] N. Hayazawa, K. Furusawa, S. Kawata, Nanometric locking of the tight focus for optical microscopy and tip-enhanced microscopy. Nanotechnology 23, 465203 (2012). 23092852 10.1088/0957-4484/23/46/465203

[40] M. Abe, Y. Sugimoto, T. Namikawa, K. Morita, N. Oyabu, S. Morita, Drift-compensated data acquisition performed at room temperature with frequency modulation atomic force microscopy. Appl. Phys. Lett. 90, 203103 (2007).

[41] E. E. Moon, H. I. Smith, Nanometer-precision pattern registration for scanning-probe lithographies using interferometric-spatial-phase imaging. J. Vac. Sci. Technol. B 24, 3083–3087 (2006).

[42] T. Yano, T. Ichimura, S. Kuwahara, P. Verma, S. Kawata, Subnanometric stabilization of plasmon-enhanced optical microscopy. Nanotechnology 23, 205503 (2012). 22543309 10.1088/0957-4484/23/20/205503

[43] T. Yano, Y. Tsuchimoto, M. Mochizuki, T. Hayashi, M. Hara, Laser scanning-assisted tip-enhanced optical microscopy for robust optical nanospectroscopy. Appl. Spectrosc. 70, 1239–1243 (2016). 27412187 10.1177/0003702816652369

[44] R. Kato, Y. Saito, P. Verma, Near-field absorption imaging by a Raman nano-light source. RSC Adv. 6, 113139–113143 (2016).

[45] S. B. Anantharaman, K. Jo, D. Jariwala, Exciton-photonics: From fundamental science to applications. ACS Nano 15, 12628–12654 (2021). 34310122 10.1021/acsnano.1c02204

[46] T. Chowdhury, K. Jo, S. B. Anantharaman, T. H. Brintlinger, D. Jariwala, T. J. Kempa, Anomalous room-temperature photoluminescence from nanostrained MoSe_2_ monolayers. ACS Photonics 8, 2220–2226 (2021).

[47] J. C. Kotsakidis, Q. Zhang, A. L. Vazquez De Parga, M. Currie, K. Helmerson, D. K. Gaskill, M. S. Fuhrer, Oxidation of monolayer WS_2_ in ambient is a photoinduced process. Nano Lett. 19, 5205–5215 (2019). 31287707 10.1021/acs.nanolett.9b01599

[48] Q. Wang, Q. Zhang, X. Zhao, Y. J. Zheng, J. Wang, X. Luo, J. Dan, R. Zhu, Q. Liang, L. Zhang, P. K. J. Wong, X. He, Y. L. Huang, X. Wang, S. J. Pennycook, G. Eda, A. T. S. Wee, High-energy gain upconversion in monolayer tungsten disulfide photodetectors. Nano Lett. 19, 5595–5603 (2019). 31241969 10.1021/acs.nanolett.9b02136

[49] C. Lee, B. G. Jeong, S. J. Yun, Y. H. Lee, S. M. Lee, M. S. Jeong, Unveiling defect-related raman mode of monolayer WS_2_ via tip-enhanced resonance Raman scattering. ACS Nano 12, 9982–9990 (2018). 30142265 10.1021/acsnano.8b04265

[50] W. Wang, F. Shao, M. Kröger, R. Zenobi, A. D. Schlüter, Structure elucidation of 2D polymer monolayers based on crystallization estimates derived from tip-enhanced Raman spectroscopy (TERS) polymerization conversion data. J. Am. Chem. Soc. 141, 9867–9871 (2019). 31244135 10.1021/jacs.9b01765

[51] N. Kumar, S. J. Spencer, D. Imbraguglio, A. M. Rossi, A. J. Wain, B. M. Weckhuysen, D. Roy, Extending the plasmonic lifetime of tip-enhanced Raman spectroscopy probes. Phys. Chem. Chem. Phys. 18, 13710–13716 (2016). 27140329 10.1039/c6cp01641c

[52] R. Kato, M. Uesugi, Y. Komatsu, F. Okamoto, T. Tanaka, F. Kitawaki, T. A. Yano, Highly stable polymer coating on silver nanoparticles for efficient plasmonic enhancement of fluorescence. ACS Omega 7, 4286–4292 (2022). 35155921 10.1021/acsomega.1c06010 PMC8829869

[53] V. Carozo, Y. Wang, K. Fujisawa, B. R. Carvalho, A. McCreary, S. Feng, Z. Lin, C. Zhou, N. Perea-López, A. L. Elías, B. Kabius, V. H. Crespi, M. Terrones, Optical identification of sulfur vacancies: Bound excitons at the edges of monolayer tungsten disulfide. Sci. Adv. 3, 1–10 (2017). 10.1126/sciadv.1602813 PMC5409454 28508048

[54] H. Lee, G. H. Kim, J. H. Lee, N. H. Kim, J. M. Nam, Y. D. Suh, Quantitative plasmon mode and surface-enhanced raman scattering analyses of strongly coupled plasmonic nanotrimers with diverse geometries. Nano Lett. 15, 4628–4636 (2015). 26075353 10.1021/acs.nanolett.5b01322

[55] J. W. Oh, D. K. Lim, G. H. Kim, Y. D. Suh, J. M. Nam, Thiolated DNA-based chemistry and control in the structure and optical properties of plasmonic nanoparticles with ultrasmall interior nanogap. J. Am. Chem. Soc. 136, 14052–14059 (2014). 25198151 10.1021/ja504270d

[56] J. H. Lee, J. W. Oh, S. H. Nam, Y. S. Cha, G. H. Kim, W. K. Rhim, N. H. Kim, J. Kim, S. W. Han, Y. D. Suh, J. M. Nam, Synthesis, optical properties, and multiplexed Raman bio-imaging of surface roughness-controlled nanobridged nanogap particles. Small 12, 4726–4734 (2016). 27028989 10.1002/smll.201600289

[57] C. Lee, H. Yan, L. Brus, T. Heinz, J. Hone, S. Ryu, Anomalous lattice vibrations of single-and few-layer MoS_2_. ACS Nano 4, 2695–2700 (2010). 20392077 10.1021/nn1003937

[58] R. Kato, K. Taguchi, R. Yadav, T. Umakoshi, P. Verma, One-side metal-coated pyramidal cantilever tips for highly reproducible tip-enhanced Raman spectroscopy. Nanotechnology 31, 335207 (2020). 32375128 10.1088/1361-6528/ab90b6

[59] R. A. Maniyara, D. Rodrigo, R. Yu, J. Canet-Ferrer, D. S. Ghosh, R. Yongsunthon, D. E. Baker, A. Rezikyan, F. J. García de Abajo, V. Pruneri, Tunable plasmons in ultrathin metal films. Nat. Photonics 13, 328–333 (2019).

