# Supplementary material for: Ultrastable tip-enhanced hyperspectral optical nanoimaging for defect analysis of large-sized WS2 layers
Source: Sci Adv. 2022 Jul 15;8(28):eabo4021. doi: 10.1126/sciadv.abo4021 (PMC9286508; doi:10.1126/sciadv.abo4021)
Supplement: Supplementary file 1 — Supplementary Materials Figs. S1 to S7 [file sciadv.abo4021_sm.pdf]

Science Advances
AAAS

Supplementary Materials for

Ultrastable tip-enhanced hyperspectral optical nanoimaging for defect analysis of large-sized WS_2 layers

Ryo Kato *et al.*

Corresponding author: Takayuki Umakoshi, umakoshi@ap.eng.osaka-u.ac.jp;
Prabhat Verma, verma@ap.eng.osaka-u.ac.jp

Sci. Adv. **8**, eabo4021 (2022)
DOI: 10.1126/sciadv.abo4021

This PDF file includes:

Supplementary Materials
Figs. S1 to S7

Supplementary Materials

Dependence of TERS signal intensity with 5 nm fluctuation of focus position of incident laser, the dependence of localization precision of the lateral drift correction system on time interval of drift compensation, normal Raman spectrum of bulk WS_2 and fitted TERS spectra of 2-, 3-, and 4-layered WS_2, comparison of far-field and near-field Raman spectra to provide estimation of TERS contrast and enhancement factor, TERS spectra of WS_2 showing enhancement of near-field optical signal recorded from the nanoscale protrusions, TERS spectra of WS_2 showing D- and D'-bands, TERS spectra of WS_2 from the fast and last few line scans in TERS images. SEM image of a typical silver-coated AFM cantilever tip, and illustration of experimental setup.

Our focus drift compensation system allows the focus position along the optical axis to be stabilized with the accuracy of 5 nm. To show that the 5 nm fluctuation of the focus position does not cause significant signal loss in TERS measurement, we have investigated scattering images of the metallic tip and TERS spectra of a self-assembled monolayer (SAM) of bi-phenyl-4-thiol (BPT) on a gold thin film. BPT is not easily photodegraded unlike WS_2, which could exclude the possible effect of photodegradation on loss of TERS signal, so that the dependence of focus position on TERS signal intensity can be correctly evaluated. BPT was dissolved in dimethylformamide (DMF, 99.8%, Sigma Aldrich). A gold thin film deposited on a clean glass substrate was immersed in 1 mM solution of BPT for 2 hours and the sample was subsequently rinsed by DMF first, and then ethanol to remove excess of BPT molecules. Figure S1(a-c) shows scattering images when the metallic nano-tip was in the vicinity of the sample surface and (b) the incident laser focused on the sample surface (0 nm) and it was slightly shifted by (a) -10 nm and (c) 10 nm from the sample surface, respectively. These images show that 5 nm fluctuation of the focus position along the optical axis does not degrade the scattering images of the metallic tip. Figure S1(d) shows TERS spectra of BPT with different focusing position. One can see that the 5 nm fluctuation of the focus position does not cause significant signal loss in TERS measurement.

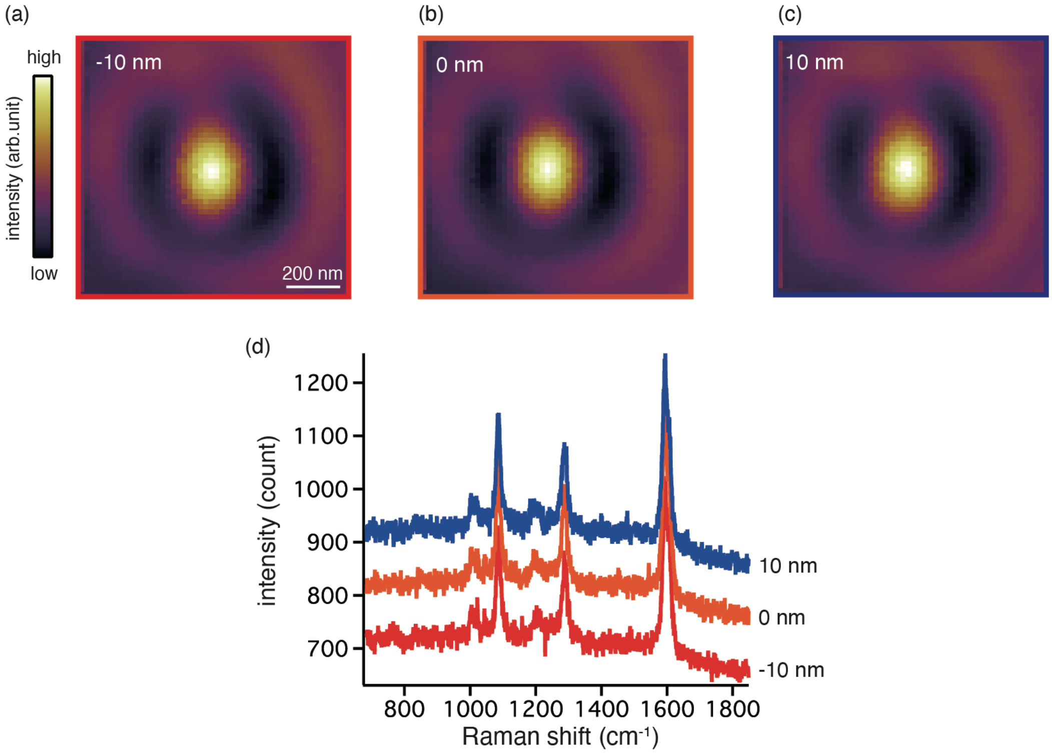

Figure S1 Scattering imaging of a metallic tip and TERS measurement with different focal positions. (a-c) Scattering images of a metallic tip-apex (a) when the laser focus is shifted by -10 nm from the substrate, (b) when the laser is properly focused on the substrate and (c) when the laser focus is shifted by 10 nm from the substrate. (d) TERS spectra of BPT with the different focus positions along the optical axis. Acquisition time of TERS spectra: 3 s. Laser power was 200 μW.

The localization precision of the relative position between the tip and the laser spot depends on duration between drift correction process, which we termed as compensation time interval. Therefore, we investigated the dependence of localization precision on compensation time interval by measuring the displacement of the relative position between the tip and the laser spot with different compensation time intervals of 10, 30 and 50 second, as shown in Figures S2(a-c). One can see that as the compensation time interval increases, the deviation of the displacement of the relative position becomes larger. Localization precision of the lateral focus drift correction system is defined as the deviation of the displacement of the relative positions. Figure S2(d) shows the dependence of localization precision on compensation time intervals. For higher localization precision of the relative position, a short time interval is better, while it increases the total measurement time. In long-time

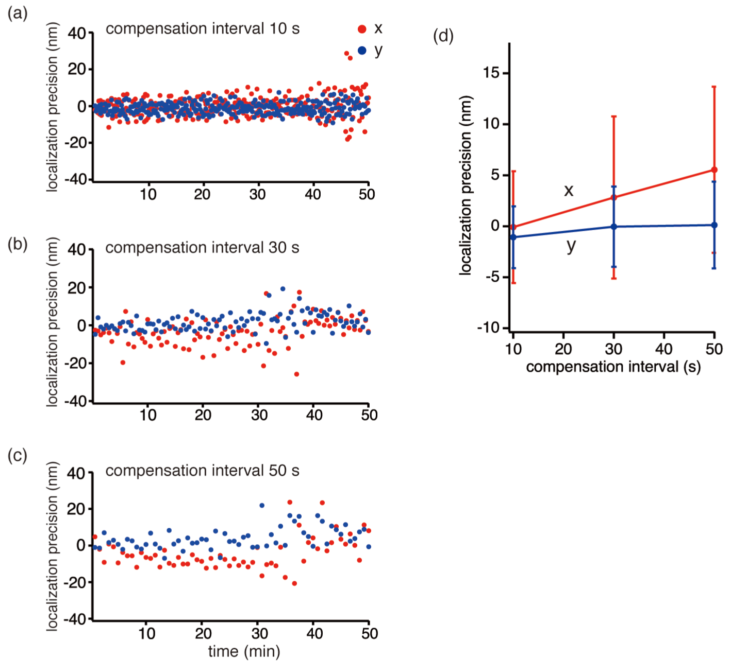

Figure S2 Investigation of localization precision the relative position between the tip and the laser spot. The displacement of the relative position between the tip and laser focus for 50 min with compensation time intervals of 10 s (a), 30 s (b) and 50 s (c). (d) The dependence of localization precision on compensation time interval.

TERS imaging, we have used compensation time interval of 30 second that always localizes the relative position between the tip and the laser spot within 10 nm.

Normal Raman spectrum of bulk WS_2 was acquired by the same TERS setup (Figure S3(a)). TERS spectra of WS_2 recorded from the areas A, B, and C marked in Figure 5(a) were processed by Lorentzian fitting to precisely evaluate the frequency shift of the Raman modes and shown in Figure S3(b). Raman shift of A_{1g} mode is sensitive to the number of WS_2 layers, while E^1_{2g} mode is not sensitive. The number of layers at the areas A, B, and C is estimated to be 2, 3, and 4 layers based on the frequency of the A_{1g} mode.

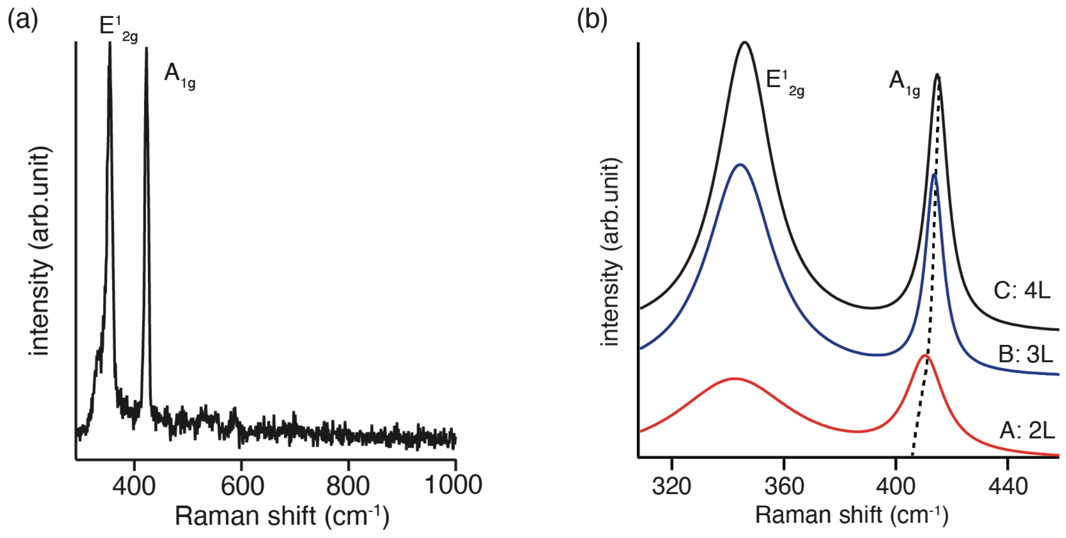

Figure S3 Far-field Raman measurement of WS_2 and TERS analysis of the number of WS_2 layers. (a) Typical far-field Raman spectrum of bulk WS_2. (b) Lorentzian-fitted TERS spectra of WS_2 measured at the areas A, B, and C in Figure 5(a). The dotted line is a guide to the eye only.

Figure S4(a) shows the comparison between near-field and far-field Raman spectra of WS_2, which allows us to estimate TERS contrast and the enhancement factor (EF) in our TERS measurement. TERS contrast and the enhancement factor are estimated by the following equations:

$$Contrast = \frac{I_{NF}}{I_{FF}} - 1, \qquad EF_{TERS} = contrast \times \frac{A_{FF}}{A_{NF}}$$

In these equations, I_{NF} and I_{FF} are the intensity of Raman signal when the TERS tip is in contact and retracted from the sample surface, respectively. A_{NF} and A_{FF} are the sample area exposed under the near-field and the far-field incident light, respectively. TERS contrast and EF in our TERS measurement are estimated to be 15.7 and 1.4×10^3, respectively, where the wavelength of the incident light is 638 nm and the size of the near-field light was regarded as 10 nm by taking the spatial resolution of our TERS imaging into consideration.

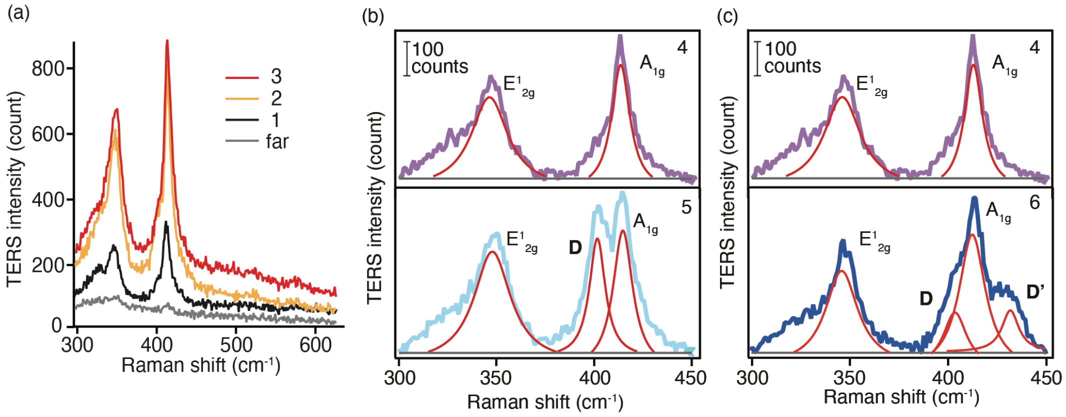

Figure S4 TERS and far-field Raman spectral analysis. (a) TERS spectra of the WS_2 monolayer measured at the area 1, 2 and 3 marked in Figure 6(a). Far-field Raman spectrum of WS_2 recorded at basal plane was also shown. (b) TERS spectra extracted from two different points in Figure 6(a) marked by 4 and 5, respectively. The red curves in the spectra show the fitted Lorentzian peaks and gray lines are baselines for fitting. (c) TERS spectra extracted from two different points in Figure 6(a) marked by 4 and 6.

5 TERS spectra of WS_2 layers obtained from different locations within the first several line scans and 5 TERS spectra obtained from different locations within the last several line scans in Figure 6(c) are shown in Figures S5 (a) and S5 (b), respectively. Also, the averaged values and the standard deviations of TERS intensity (A_{1g} mode) obtained from these 5 TERS spectra are shown in Figure S5(c). It should be noted that we chose the areas for constructing the averaged spectra, where no significant defect-related TERS signal or strong enhancement of photoluminescence signal was observed for properly evaluating the stability of our TERS system.

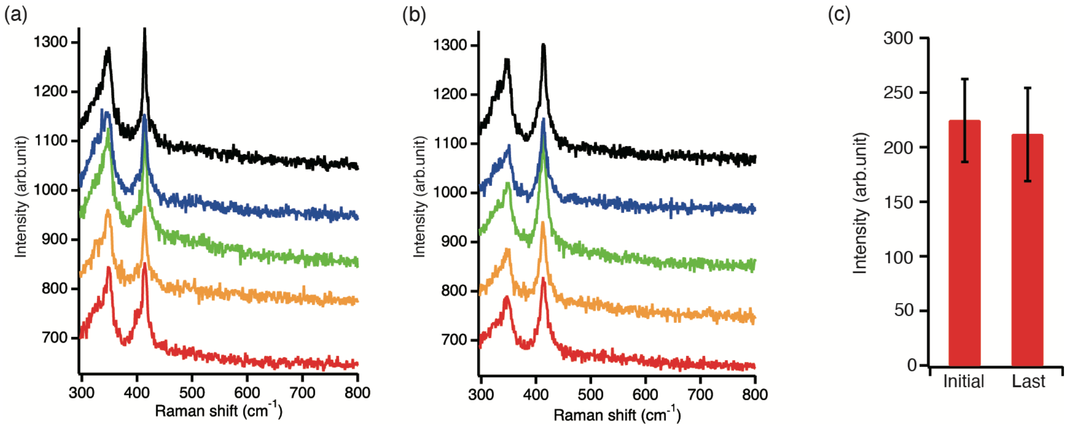

Figure S5 Stabilized TERS intensities during long-time TERS imaging. (a), (b) The comparison of averaged TERS spectra obtained from different locations within the initial several line scans and the last several line scans of the TERS image shown in Figure 6(c). (c) The averaged values and the standard deviations of TERS intensity obtained from 5 the TERS spectra.

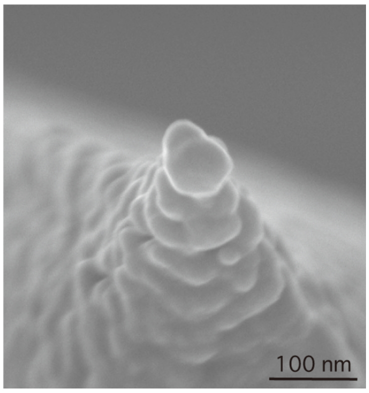

Figure S6 Scanning electron micrograph around the apex of a typical silver-coated TERS tip used for long-time TERS imaging. The structure of the apex of TERS tips showing strong signal enhancement was observed by a scanning electron microscope (SEM, SU9000, Hitachi). The SEM image of the apex of one of the typical TERS tips that we used is shown in Figure S5. The apex of the TERS tip possesses a single metallic nanoparticle responsible for electromagnetic field enhancement.

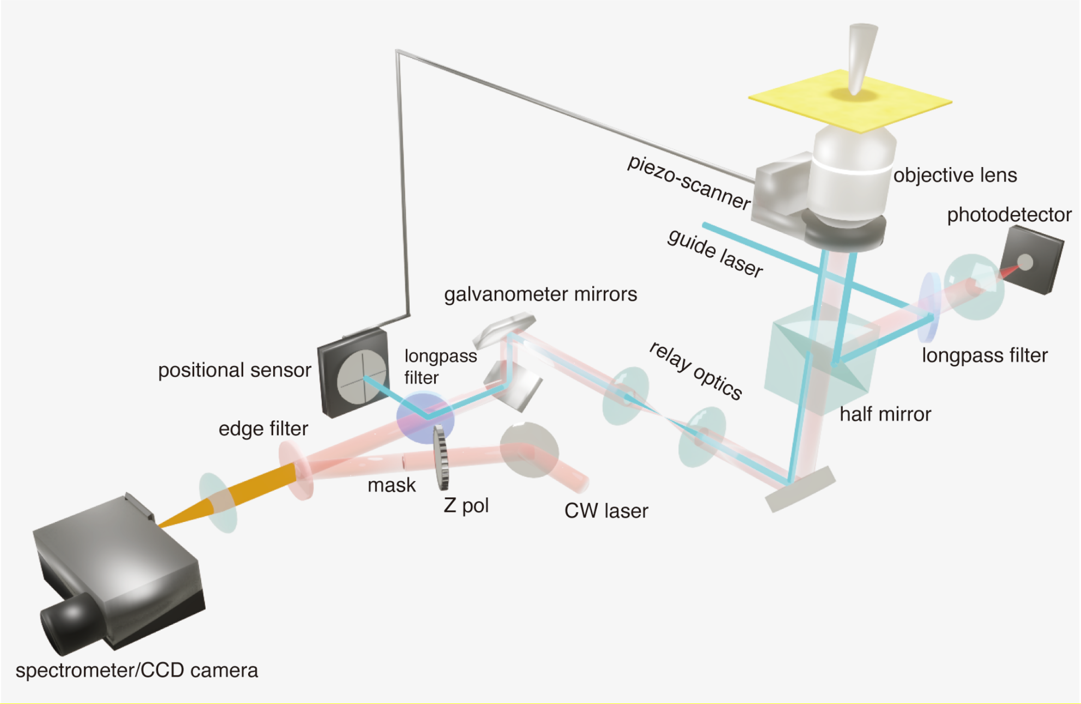

Figure S7 Schematic illustration of our TERS setup for long-time TERS imaging. The figure shows a schematic illustration of our TERS setup. The spectrometer coupled with the CCD camera used for Raman measurement, the piezo-scanner for focus drift compensation, and the photodetector and galvanometer mirrors used for tip drift compensation were synchronized by a home-made Igor program and LabVIEW.
